# Systemic Transplantation of Human Umbilical Cord Derived Mesenchymal Stem Cells-Educated T Regulatory Cells Improved the Impaired Cognition in AβPPswe/PS1dE9 Transgenic Mice

**DOI:** 10.1371/journal.pone.0069129

**Published:** 2013-07-25

**Authors:** Hongna Yang, Hui Yang, Zhaohong Xie, Lifei Wei, Jianzhong Bi

**Affiliations:** Department of Neural Medicine, the Second Hospital of Shandong University, Jinan City, Shandong Province, China; Children's Hospital Boston/Harvard Medical School, United States of America

## Abstract

Alzheimer’s disease (AD) is one of most prevalent dementias, which is characterized by the deposition of extracellular amyloid-beta protein (Aβ) and the formation of neurofibrillary tangles within neurons. Although stereotaxic transplantation of mesenchymal stem cells (MSCs) into the hippocampus of AD animal model as immunomodulatory cells has been suggested as a potential therapeutic approach to prevent the progress of AD, it is invasive and difficult for clinical perform. Systemic and central nervous system inflammation play an important role in pathogenesis of AD. T regulatory cells (Tregs) play a crucial role in maintaining systemic immune homeostasis, indicating that transplantation of Tregs could prevent the progress of the inflammation. In this study, we aimed to evaluate whether systemic transplantation of purified autologous Tregs from spleens of AβPPswe/PS1dE9 double-transgenic mice after MSCs from human umbilical cords (UC-MSCs) education *in vitro* for 3 days could improve the neuropathology and cognition deficits in AβPPswe/PS1dE9 double-transgenic mice. We observed that systemic transplantation of autologous Tregs significantly ameliorate the impaired cognition and reduced the Aβ plaque deposition and the levels of soluble Aβ, accompanied with significantly decreased levels of activated microglia and systemic inflammatory factors. In conclusion, systemic transplantation of autologous Tregs may be an effective and safe intervention to prevent the progress of AD.

## Introduction

Recently, stereotaxic transplantation of mesenchymal stem cells (MSCs) as a group of multipotent stem cells and immunosuppressive cells into the bilateral hippocampus of Alzheimer’s disease (AD) animal model was considered to be an effective method to prevent the progress of AD by modulation of central nervous systemic inflammation [1–3]. However, stereotaxic transplantation is an invasive method and difficult for clinical perform. Alzheimer’s disease is the most common cause of dementia beginning with impaired memory, which accounts for about 60% of dementia cases. It has been estimated that about 35.6 million people lived with dementia in 2010, with 4.6 million new cases arising every year [4,5]. The etiology of Alzheimer’s disease, whose neuropathology is characterized by the deposition of extracellular amyloid beta protein (Aβ) and neurofibrillary tangle formation within neurons, remains unclear [6]. It has been hypothesized that the imbalance of the production and degradation of Aβ protein is considered to be the principal initiating factor. Now, accumulating evidences suggest that inflammation may play an important role in the pathogenesis of AD [7,8]. It has been reported that anti-inflammation drugs can improve the impairment of cognition [9–11]. In addition, the incidence of AD in patients treated with nonsteroidal anti-inflammation drugs can be decreased [12]. T regulatory cells (Tregs) characterized CD4^+^ T cells expressing CD25 (the interleukin-2 (IL-2) receptor α-chain), which were first proposed and confirmed in mice in the early 1970s, play an important role in maintaining the immune homeostasis and self-tolerance through regulating the ratio of Th1/Th2 cells and secretion of immunosuppressive cytokines interleukin-10 (IL-10) and/or transforming growth factor-β1 (TGF-β1) [13–16]. Recently, some scientists proposed that transplantation of *ex vivo* expanded Tregs not only prevented the progression of ongoing inflammatory and autoimmune diseases in mice but also inhibited the occurrence of graft-versus-host disease after bone marrow transplantation [17]. In addition, it was reported that transplantation of autologous peripheral lymphocytes after human cord blood stem cells education *in vitro* could reverse the progress of T1D in clinical trial [18]. Recently, evidences indicated that abnormalities of Tregs in cell number and/or function were associated with the inflammation or pathogenesis of AD [19]. More important, it was reported that Tregs also suppressed the characteristic glial response to injury in the CNS, assumed to be destructive to neuronal survival [20].

MSCs as multipotent nonhematopoietic progenitor cells are capable of differentiating into various lineages including osteoblasts, chondrocytes and adipocytes. In recent years, MSCs from human umbilical cord blood and bone marrow have been extensively investigated as immunomodulatory and regenerative cells *in vitro* and *in vivo*. It has been confirmed that MSCs from bone marrow and/or human umbilical cord blood display an important immunomodulatory capability *via* inhibiting the proliferation and function of T cells, B cells and natural killer (NK) cells as well as the function of mature monocytes-derived dendritic cells *in vitro* [21–23]. In addition, MSCs from bone marrow and/or human umbilical cord blood as immunomodulatory cells *in vivo* have been used to prevent the progression of the autoimmune and inflammatory diseases, i.e. multiple sclerosis (MS), type i diabetes (T1D), chronic colitis and experimental autoimmune uveitis via inducing the production of Tregs *in vivo* and/or reducing the production of pro-inflammatory factors as well as improving the production of anti-inflammatory factors [23–27] [28]. It also has been confirmed that MSCs from bone marrow and/or human umbilical cord blood can induce the phenotype expression of Treg cells or recruit Treg cells in peripheral lymphocytes *in vitro* [24,29]. Human umbilical cords as the clinical waste provide an alternative source for isolating plenty of MSCs [30]. It does no harm to donors and we can easily get plenty of MSCs from umbilical cords. More and more evidences demonstrate that MSCs from human umbilical cords (UC-MSCs) have the similar immuonomodulatory function as MSCs from bone marrow [31–33].

Based on these previous findings, we successfully isolated MSCs from umbilical cords. We tried to confirm whether UC-MSCs can modulate the frequency and/or function of Tregs *in vitro*. In addition, we aimed to investigate whether systemic transplantation of Tregs after UC-MSCs education *in vitro* could improve the impaired cognition of AβPPswe/PS1dE9 transgenic mice, an animal model of AD. It is the first time to propose that autologous transplantation of purified Tregs after UC-MSCs education is used to prevent the progress of neurodegenerative diseases, such as AD.

## Methods and Materials

### Mice

Heterozygous AβPPswe/PS1dE9 double transgenic (Tg) mice (n=40) and C57BL6 mice (n=15) as wild type (WT) control (male, 6 months old) were obtained from Beijing HFK Bio-Technology, Institute of Laboratory Animal Science, Chinese Academy of Medical Science (Beijing, China) and used throughout the study. The animals were housed in temperature- and humidity-controlled rooms and on a 12h/12h light/dark cycle. All the animal protocols and procedures described in this study were approved and in accordance with the guidelines of the Ethical Committee for Animal Experiments of Shandong University.

### Human umbilical cord derived mesenchymal stem cells (UC-MSCs)

Umbilical cords were obtained under sterile conditions from full-term infants delivered by caesarean section from obstetrical department of the second hospital of Shandong University with donors’ written informed consent. Human tissue collection for research was approved by the institutional review board of the Shandong University and the Second Hospital of Shandong University. MSCs were isolated from umbilical cord according to the protocol [31,34]. In brief, the cords were washed by PBS. The vessels were removed to retain the Wharton’s jelly. The Wharton’s jelly was cut into 1mm^3^ pieces and then put the pieces on the bottom of tissue culture dishes for two hours at 37°C and 5% carbon dioxide incubator, then added about 15ml medium containing DMEM (low glucose) supplemented with 10% fetal bovine serum (FBS, Invitrogen), 1% L-glutamine and 1% Penicillin-Streptomycin for 7 days at 37°C and 5% carbon dioxide incubator. After 7 days, the pieces were removed and the primary cells were passaged by 1-min treatment with 0.25% trypsin and 0.02% EDTA at 37°C. The cell culture was maintained at 37°C in an incubator with 5% (v/v) CO_2_. The medium was changed every 3 days. Umbilical cord-derived MSCs were passaged when reached 90% confluences by 1-min treatment with 0.25% trypsin and 0.02% EDTA at 37°C. All UC-MSCs used in the experiment were controlled within passage 3-6.

### UC-MSCs co-cultured with spleen lymphocytes

Spleen lymphocytes were isolated from the spleens of Tg mice according to the protocol [35]. In brief, the spleens were removed from AβPPswe/PS1dE9 double mice (n=10) of 6 months age. Single cell suspensions were made by mincing and grinding the spleen through a 40-µm nylon cell strainer (Coring, USA). Mononuclear cells were harvested using mouse spleenocyte separation medium (Dakewe, China). The spleen lymphocytes were cultured in advanced RPMI 1640 supplemented with 10% FBS, 1% L-glutamine and 1% Penicillin-Streptomycin. UC-MSCs (1×10^5^) were plated on the 12-well plate overnight. The lymphocytes were co-cultured in the 12-well plate at the density of 5×10^5^/well/ml with UC-MSCs at the ratio of 1:5 (UC-MSCs: spleen lymphocytes) or without UC-MSCs in the medium for spleen lymphocytes *in vitro* for 3 days. Each experiment was performed in triplicate.

### Flow analysis

Flow analysis was performed according to the protocol described by Yong Zhao [24]. The antibodies used in the experiments were: Anti-Mouse APC-conjugated CD4 and Anti-Mouse PE-conjugated CD25 (eBioscience, USA). For flow analysis, the suspending lymphocytes were firstly harvested from co-culture medium by centrifugation, then washed with PBS with 0.2% FBS. After washing, the suspending cells were incubated with antibodies at 4 °C for 30 min. After counting the number of cells, the cells were washed with cold PBS prior to flow analysis.

### Isolation of CD4^+^CD25^+^ T regulatory cells

The spleen lymphocytes after with or without UC-MSCs education for 3 days *in vitro* were harvested for isolation of CD4^+^CD25^+^ T regulatory cells using MACS cell separation with CD4^+^CD25^+^ Regulatory T Cell Isolation Kit mouse (Miltenyi Biotec, USA). The isolation protocol was according to the manufacturer’s protocol. We could obtain the purity of the isolated CD4^+^CD25^+^ T regulatory cells was more than 98% [36].

### T regulatory cells co-cultured with CFSE labeling spleen lymphocytes

The purified T regulatory cells (1×10^4^cells/well) after UC-MSCs education or without UC-MSCs education co-cultured with spleen lymphocytes (1×10^5^cells/well) at the ratio 1:10 in the medium for the spleen lymphocytes for 3 days. The spleen lymphocytes were labeled by CFSE (Invitrogen, USA) according to the protocol described by Ben before co-culture with purified Treg cells [37]. Briefly, 10^6^/ml lymphocytes were suspended in 10µM CFSE with PBS and 0.1% BSA for 10min at 37°C incubator. After incubation, the cells were washed twice with cold medium. After labeling, the CFSE-labeled cells were co-cultured in the 48 well-plate with the purified CD4^+^CD25^+^ T regulatory cells after with UC-MSCs education or without UC-MSCs education, at the density of 5×10^5^/well/*ml* with 10 *ug*/*ml* PHA (Sigma, USA). The suspending cells were harvested and used for the flow analysis at 488 nm excitation.

### Cell transplantation

The purified CD4^+^CD25^+^ T regulatory cells from Tg mice spleen lymphocytes after with UC-MSCs education were administered to Tg mice (n=15) by intracardiac injection at the dose of 0.5×10^6^ cells/100 µl PBS for the first time, followed by a second injection at the dose of 0.2×10^6^ cells/100 µl PBS one week later. Another Tg mice (n=15) were injected the same volume of PBS as the control. Two weeks after the second injection of CD4^+^CD25^+^ T regulatory cells, the mice were performed at behavioral test.

### Behavioral experiment

Behavioral experiment was performed on Tg mice at three weeks after the initial injection of CD4^+^CD25^+^ T regulatory cells (n=15) or PBS (n=15) and C57BL6 mice of same age (n=15) as control. Morris water maze (MWM) test was conducted to evaluate spatial memory performance in these animals. Detailed methodology is previously described by Vorhees CV [38]. In brief, the pool (1.2m diameter) was sited in a well-light room (24°C), and distinct visual cues were placed on the walls of the pool. The mice were released at four different start point (N, E, SE, NW) every day for the next consecutive 5 days to find the hidden platform (located under the water 0.5cm, in the center of SW quadrant) under the water level after the first day of habitation. If the mice cannot successfully find the hidden platform in 60s, we gently guided the mice to the platform for 15s. 24h after the final day of training, the hidden platform was removed during the probe test and the mice were released into the water level at the NE point. A digital pick-up camera was used to record the latency and monitor the animals’ behavior, including escape latency, the number of platform crossing and the time in the target quadrant. A computer program was used for data analysis (ZH0065, Zhenhua Bioequipments, China) for the trials.

### Immunohistochemistry

At the end of behavioral tests, 6 mice from each group were killed by perfusion with 0.9% saline solution followed by 4% paraformaldehyde in PBS (pH 7.4), and the brains were removed and cut into 10µm sections for Thioflavin S (Sigma, USA) staining (for assessing the area of Aβ plaque) and immunohistochemistry staining. The brains were frozen in Tissuse-Tek embedding compound (Sakura Finetek, Japan) and sectioned on a cryostat (Leica CM1850, Germany). The method of Thioflavin S staining and Iba-1 immunofluorescent staining followed the protocol previously descried by Fiorentini [39]. Ten serial sections at an interval of every 5^th^ section throughout frontal cortex or hippocampus for Thioflavin S staining were incubated for 5 min at a concentration of 0.5% thioflavin S dissolved in 50% ethanol, and then washed twice with 50% ethanol for 5 min each and once with distilled water for 5 min, and mounted with mounting medium for fluorescent (Vector, USA). Green fluorescence stained plaques were observed using fluorescence microscopy (Olympus). Ten serial sections for Iba-1 immunofluorescent staining were treated with 0.4% Triton X-100 in PBS for 30 min at room temperature (RT). After rinsing with PBS, three times, the sections were incubated with 5% goat serum (Jackson ImmunoResearch Laboratories) in PBS for 1 h. They were then incubated with the primary antibodies (rabbit anti-Iba-11:800, Wako, Japan) overnight at 4°C. After rinsing in PBS, the sections were incubated with secondary antibody for 1h at RT. The corresponding 2^nd^ antibodies were: TRITC goat anti-rabbit IgG (1:200 Jackson ImmunoResearch Laboratories, USA). After rinsing, the sections were mounted with mounting medium for fluorescence (Vector, USA), and examined with fluorescence microscopy (Olympus, Japan). The percent of Iba-1 positive cells and area of Aβ plaque occupied the whole slice were autoanalyzed by the software IPP6.0.

### ELISA

The remaining mice (n=9) were killed by over dose 10% chloral hydrate (4ml/kg weighht, i.p.). Before the mice died, the peripheral blood was harvest in the tube with heparin sodium to prevent blood from agglutination. We got the plasma for testing cytokine after centrifugation after centrifugation (800 rpm/min, 5mins) and stored at -80°C. The cytokine IL-10, TGFβ1 and IFN-γ were measured by ELISA kits (eBioscience, USA) according to the manufacturer’s recommendation. After the mice died, we immediately removed the brain on ice and transferred the brains to -80°C refrigerator for storage. We obtained the whole brain tissue homogenization according to according to the manufacturer’s recommendation (Aβ1-40 & Aβ1-42, Invitrogen, USA). In brief, the whole of brain tissue was placed in a 1:8 dilution of 5M ice guanidine HCl/50mM Tis HCl and thoroughly minced. The homogenate was diluted at a ratio of 1:50 with dilution buffer (PBS with 5% BSA, 0.03% Tween-20, pH7.4) containing an inhibitor protease complex and centrifuged at 16,000 rpm for 30 min at 4°C. The samples and protein standard were added t into the 96-well plate and were recorded at 450 nm using microplate reader (Biorad, USA). Each standard and experimental sample was run in duplicate and the results were averaged.

### Statistical analysis

Statistical analysis was performed using GraphPad Prism (GraphPad). Data were analyzed using two-way ANOVA and two sample t test. Data were expressed as means with SEM. Significance was set at P<0.05.

## Results

### UC-MSCs improved the frequency and function of CD4^+^CD25^+^ T regulatory cells in spleen lymphocytes from AβPPswe/PS1dE9 transgenic mice

**Figure 1:**
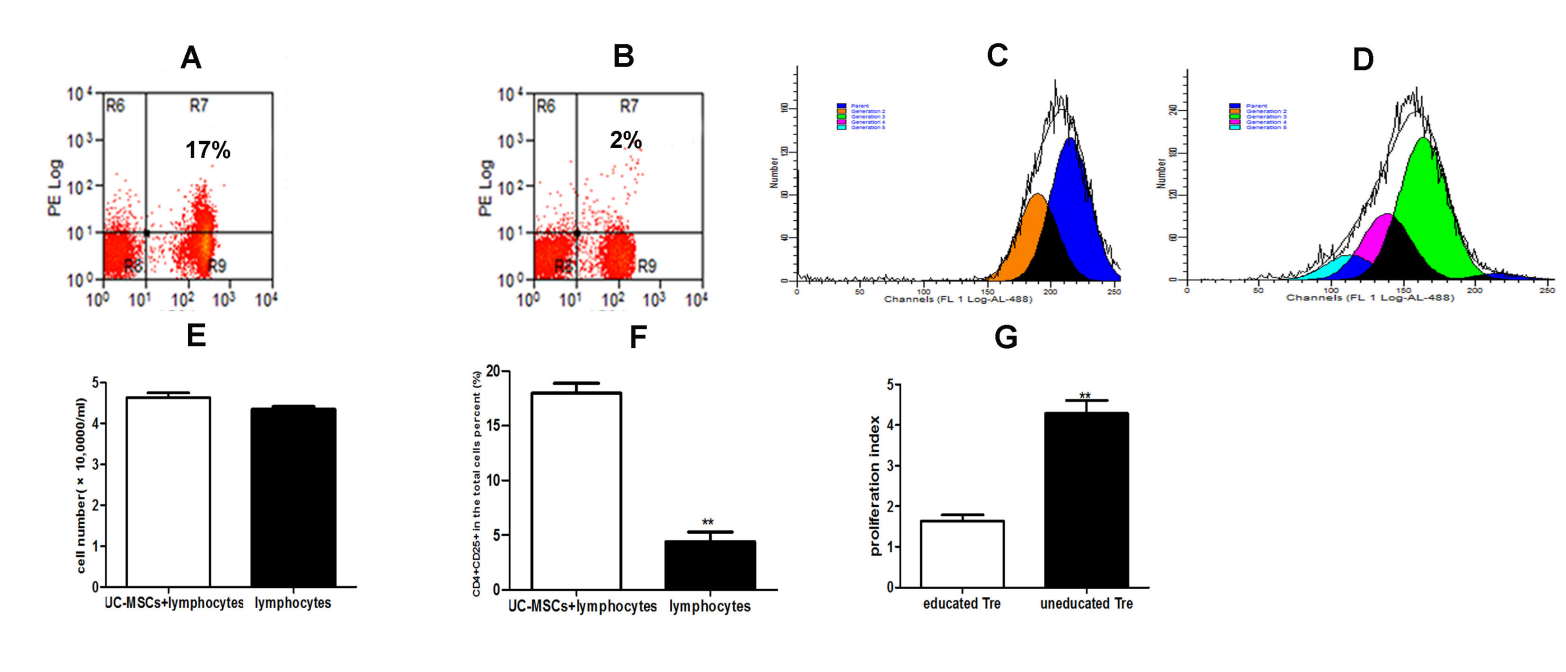
UC-MSCs improved the frequency and function of CD4^+^CD25^+^ T regulatory cells in spleen lymphocytes from Tg mice. Spleen lymphocytes (5×10^5^cells/well) were cultured in 24-well plate in the presence or absence of UC-MSCs (1×10^5^cells/well) *in vitro* for 3 days. **A & B** Representative dot plot results of APC-CD4^+^ PE-CD25^+^ T cells in the total cell population in the presence (A) and absence (B) of UC-MSCs by flow cytometry. **C & D** Representative results of CFSE label spleen lymphocytes (1×10^5^cells/well) with PHA (10µg/ml) stimulation in the presence of isolated CD4^+^CD25^+^ T cells (1×10^4^cells/well) with UC-MSCs education (C) and without UC-MSCs education (D). **E** The bar graph showing that UC-MSCs had no effect on the proliferation of spleen lymphocytes at the ratio 1:5. Data represented mean (±SD) of four experiments. **F** UC-MSCs significantly increased the frequency of CD4^+^ CD25^+^ T cells in the total spleen lymphocytes. Data represented mean (±SD) of four experiments. **p<0.01. **G** CD4^+^CD25^+^ T cells with UC-MSCs education significantly inhibited the proliferation of PHA stimulated spleen lymphocytes. Proliferation index was obtained by the software ModFit. Data represented mean (±SD) of four experiments. **p<0.01.

To investigate whether UC-MSCs exerted immunomodulation on Treg cells, we measured the frequency of Treg cells by multicolor flow cytometry. Before flow cytometry, we counted the number of the harvested suspend spleen lymphocytes in the presence and absence of UC-MSCs co-culture. As illustrated in Figure 1E, we observed that UC-MSCs had no effect in stimulating and/or inhibiting the proliferation of the resting mouse spleen lymphocytes at the ratio of 1:5 (UC-MSCs: spleen lymphocytes) by cell counting. Flow cytometry data revealed that the frequency of CD4^+^CD25^+^ T regulatory cells in the total cell population in the presence of UC-MSCs *in vitro* for 3 days was significantly increased compared to those in the absence of UC-MSCs (Figure 1A, 1B & 1F, *p*<0.01). To investigate whether Treg cells after UC-MSCs education had the immunosuppressive function, we co-cultured the purified educated and uneducated CD4^+^CD25^+^ T regulatory cells with CFSE labeling allogenic spleen lymphocytes in the presence of PHA (10*ug/ml*) *in vitro* for 3 days. After co-culture for 3 days *in vitro*, we did flow cytometry and analyzed the proliferation index by the software ModFit. We found that the purified Treg cells after UC-MSCs education significantly reduced the proliferation index of PHA stimulated spleen lymphocytes compared to those without UC-MSCs education by statistic analysis (Figure 1C, 1D & 1G, *p*<0.01). These data suggested that UC-MSCs could improve not only the frequency but also suppressive function of CD4^+^CD25^+^ T regulatory cells *in vitro*.

### Transplantation of UC-MSCs educated CD4^+^CD25^+^ T regulatory cells regulated the levels of cytokines in the plasma of AβPPswe/PS1dE9 transgenic mice

**Figure 2:**
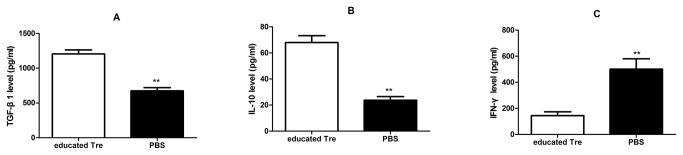
Transplantation of UC-MSCs educated CD4^+^CD25^+^ T regulatory cells regulated the levels of cytokines in the plasma of Tg mice. **A & B** Transplantation of UC-MSCs educated CD4^+^CD25^+^ T regulatory cells significantly improved the plasma level of TGFβ1 (A) and IL-10 (B). **C** Transplantation of UC-MSCs educated CD4^+^CD25^+^ T regulatory cells significantly reduced the plasma level of interferon-γ. Data represented mean (±SD) of four experiments. **p<0.01.

To examine whether CD4^+^CD25^+^ T regulatory cells after UC-MSCs education could still exert immunoregulatory function *in vivo*, we measured the levels of plasma pro-inflammatory (interferon-γ) and anti-inflammatory cytokines (IL-10 and TGFβ1) by ELISA kits at the end of Morris water maze. As illustrated in Figure 2A & 2B, we found that the plasma levels of the cytokine TGF-β1 and IL-10 were both significantly increased in the plasma of the Tg mice receiving CD4^+^CD25^+^ T regulatory cells after UC-MSCs education *in vitro* for 3 days compared to the Tg mice receiving PBS. In contrast, we observed that UC-MSCs educated CD4^+^CD25^+^ T regulatory cells exerted a significant adverse tendency in the plasma level of interferon-γ compared to those receiving PBS (Figure 2C, *p*<0.01). These data suggested that UC-MSCs educated CD4^+^CD25^+^ T regulatory could not only exerted the immunosuppressive function in vivo but also alleviate the systemic inflammation by systemic administration.

Transplantation of UC-MSCs educated CD4^+^CD25^+^ T regulatory cells not only inhibited microglia activation but also reduced the level of Aβ in the AβPPswe/PS1dE9 transgenic mice.

**Figure 3:**
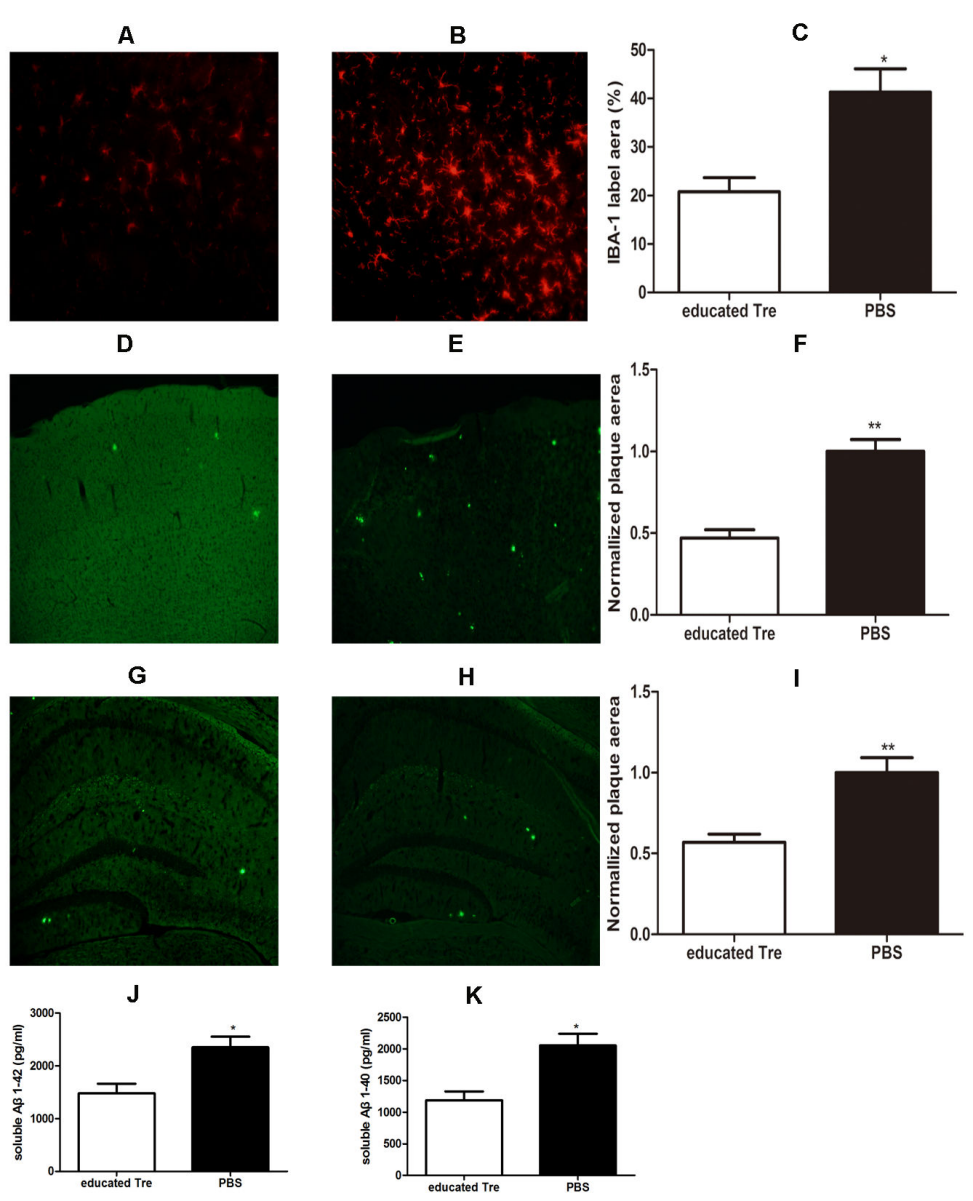
Transplantation of UC-MSCs educated CD4^+^CD25^+^ T regulatory cells inhibited microglia activation and reduced the the level of Aβ in Tg mice. Immunofluorescence using a primary antibody (Iba-1) in conjunction with TRITC-conjugated secondary antibody was used to label microglia. Thiofalvin S staining was used to label the Aβ plaque. **A & B** Representative result of activated microglia in the brain of AβPPswe/PS1dE9 transgenic mice with systemic transplantation of UC-MSCs educated CD4^+^CD25^+^ T regulatory cells (A) and PBS (B). **C** The bar showed that systemic transplantation of UC-MSCs educated CD4^+^CD25^+^ T regulatory cells significantly reduced the number of the Iba-1 positive cells in the brain of Tg mice. Data from 10 serial sections at an interval of every 5^th^ section through the bilateral cortex and hippocampus were summed to derive representative values for each animal for positive cells and 6 mice per group. Data are reported as mean ±S.E.M. *p<0.05. **D & E** Representative results of Aβ plaque in the cortex of βPPswe/PS1dE9 transgenic mice with systemic transplantation of UC-MSCs educated CD4^+^CD25^+^ T regulatory cells (D) and PBS (E). **G&H** Representative results of Aβ plaque in the hippocampus of AβPPswe/PS1dE9 transgenic mice with systemic transplantation of UC-MSCs educated CD4^+^CD25^+^ T regulatory cells (G) and PBS (H). **F&I** The bar showed that Systemic transplantation of UC-MSCs educated CD4^+^CD25^+^ T regulatory cells significantly reduced the area of Aβ plaque in the cortex (F) and hippocampus (I). Data from 10 serial sections at an interval of every 5^th^ section through the bilateral cortex and hippocampus were summed to derive representative values for each animal for total plaque area and 6 mice per group. Data are reported as mean ±S.E.M. **p<0.01. **J&K** The bar showed that systemic transplantation of UC-MSCs educated CD4^+^CD25^+^ T regulatory cells significantly reduced the level of the whole brain soluble Aβ1-42 (J) and Aβ1-40 (K) by ELISA test. Data from 6 mice are reported as mean ±S.E.M. *p<0.05.

To confirm whether systemic transplantation of UC-MSCs educated CD4^+^CD25^+^ T regulatory cells could exert similar immunoregulatory function in central nervous system as the periphery, we used IBA-1 antibody to label the microglia by flouresecent immunohistochemistry to analyze the status of microglia cells in the brain of Tg mice. We observed that most of microglia cells exerted small bodies and thin and long processes in the cortex after treatment with UC-MSCs educated CD4^+^CD25^+^ T regulatory cells, compared to those exerting enlarged cell bodies and short processes in the cortex after with PBS treatment (Figure 3A & 3B). In addition, we found that transplantation of UC-MSCs educated CD4^+^CD25^+^ T regulatory cells significantly reduced the number of activated microglia cells, whose morphology was enlarged bodies and short processes (Figure 3 C, *p*<0.05).

To test whether UC-MSCs educated CD4^+^CD25^+^ T regulatory cells have the effect on the area of Aβ plaque at the end of the fourth week of the initial cell transplantation, we measured the total area of the cortex and hippocampus by Thioflavin-S staining. In the cortex and hippocampus, statistic analysis showed that the area and the number of Aβ plaque were significantly reduced and the morphology of Aβ plaque was less loosen after transplantation of UC-MSCs educated CD4^+^CD25^+^ T regulatory cells (Figure 3D–3I, *p*<0.01). The levels of the soluble Aβ1-40 and Aβ1-42 were measured by ELISA kits. The result revealed that transplantation of UC-MSCs educated CD4^+^CD25^+^ T regulatory cells could significantly reduce the level of the total soluble Aβ1-40 and Aβ1-42 in the brain (Figure 3J & 3K, *p*<0.05).

### Transplantation of UC-MSCs educated CD4^+^CD25^+^ T regulatory improved the impairments of cognition in AβPPswe/PS1dE9 transgenic mice

**Figure 4:**
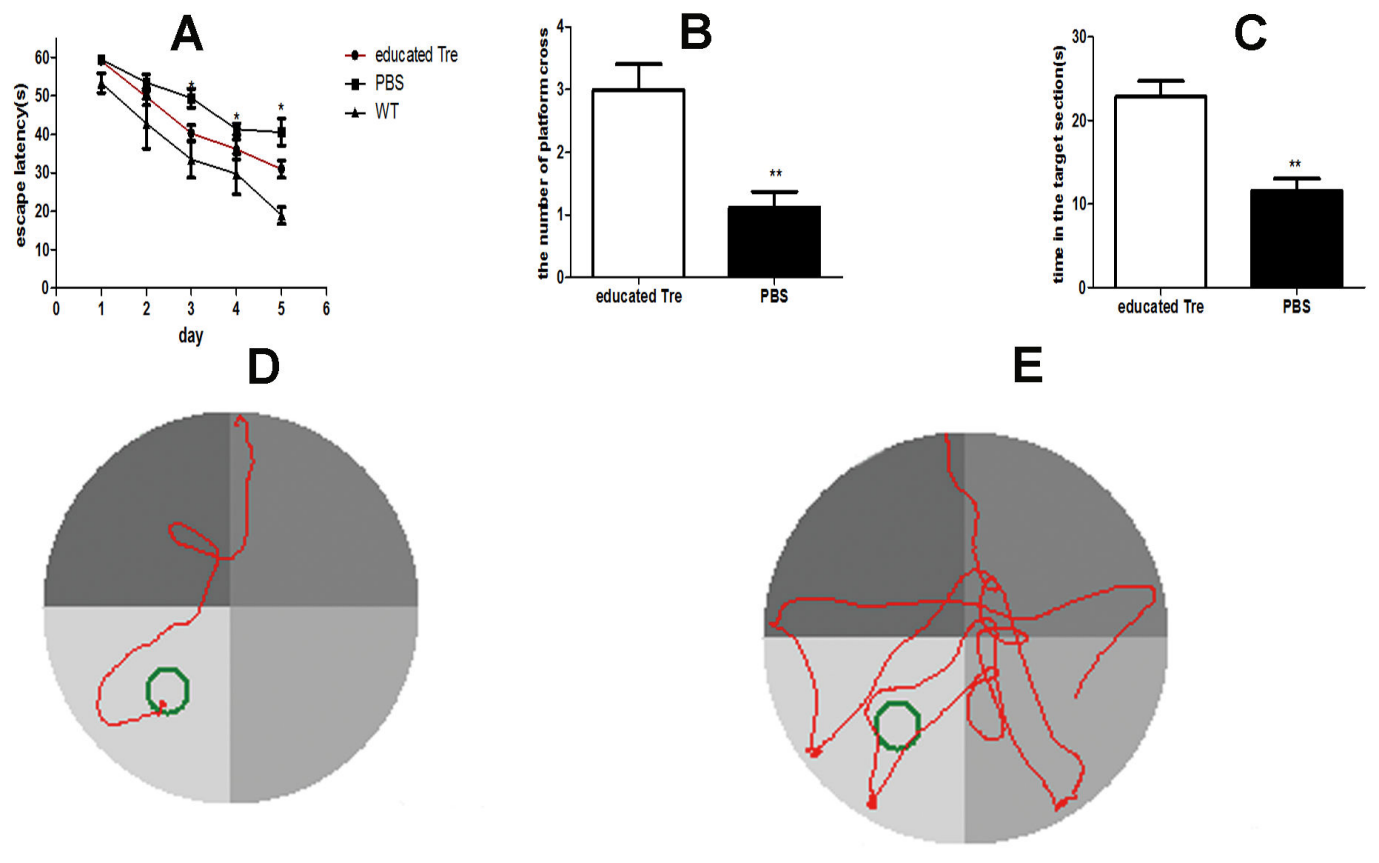
Transplantation of UC-MSCs educated CD4^+^CD25^+^ T regulatory improved the impairments of learning and memory in Tg mice. **A**. Latency to find the platform during the training was reported as mean ±S.E.M. Each point represented the mean daily values of four trials per day. The latency of the group with systemic transplantation of UC-MSCs educated CD4^+^CD25^+^ T regulatory cells was significantly lower than the group receiving vehicle after the last 3 days of training. *p＜0.05. **B&C** The bar graphs showed the number of platform location cross (B) and the time in the target section (C) during the probe trial within 60s were significantly improved in the group with systemic transplantation of UC-MSCs educated CD4^+^CD25^+^ T regulatory cells. Data are reported as mean ±S.E.M. **p<0.01. **D & E** Representative visible learning curve of transgenic mice with transplantation of UC-MSCs educated CD4^+^CD25^+^ T regulatory cells (D) and PBS (E) at day 5 of first training.

To confirm whether transplantation of UC-MSCs educated CD4^+^CD25^+^ T regulatory cells could improve the impairments of cognition, the mice were assessed the ability of learning and memory by Morris water maze at the end of the third week of the initial administration of UC-MSCs educated CD4^+^CD25^+^ T regulatory cells. The mice were trained four times from different start point to find the hidden platform in the maze for five days. We measured the ability of learning by measuring the escape latency, which was the time that the mice in the maze successfully found the hidden platform in 60s. The data showed that systemic transplantation of UC-MSCs educated CD4^+^CD25^+^ T regulatory cells significantly reduced the escape latency in the last 3 days (Figure 4A, *p*<0.05) and the pathway to find the hidden platform (Figure 4D & 4E). We also noticed although systemic transplantation UC-MSCs educated CD4^+^CD25^+^ T regulatory cells could decrease the escape latency, the transgenic mice still had the longer escape latency than the WT mice. There was no significant difference in the speed of three groups (data not show). After 24h of the last training, we removed the hidden platform and the mice were tested in probe trial for assessing the ability of memory. As illustrated in Figure 4B & 4C, we observed that transplantation of UC-MSCs educated CD4^+^CD25^+^ T regulatory cells significantly increased the number of platform crossing as well as the time in the target section during the 60s probe trial. These data indicated that systemic transplantation of UC-MSCs educated CD4^+^CD25^+^ T regulatory cells could ameliorated the cognitive impairments of AβPPswe/PS1dE9 transgenic mice.

## Discussion

AD is one of neurodegenerative diseases, which cannot be effectively cured or treated to date. Cell replacement therapy, which is considered to be an attractive method for treating the neurodegenerative diseases, such as AD and Parkinson disease (PD), is extensively investigated now. Here, we demonstrated that UC-MSCs improved not only the frequency but also the function of Tregs in vitro. More importantly, we demonstrated for the first time that systemic transplantation of purified autologous Tregs after allogeneic UC-MSCs education *in vitro* for 3 days could improve the impaired cognition and neuropathology, including reduction of Aβ plaque deposition and activated microglia as well as systemic inflammation.

In this study, we used the AβPPswe/PS1dE9 double-transgenic (Tg) mice of 6 months age as the animal model of AD, which represented the advanced stage of AD [40]. It is commonly accepted that CD4 and CD25 are used to be the markers of Tregs, which maintain the immune balance or inhibit the process of inflammation *via* several different mechanisms [16]. It has been proved that the number and/or suppressive function of Tregs in AD patients are defective [19]. Our team also found that the frequency of Tregs in Tg mice was lower than WT mice of same age (data not show). It is not new that MSCs from bone marrow and human umbilical cord blood exert the immunomodulation *in vitro and vivo* [21,23]. Recently, accumulating evidences suggested that MSCs form human umbilical cords also display immunomodulatory function by suppressing the proliferation of activated T cells *in vitro via* cell contact and/or soluble factors, or *via* converting effecter T cells into Treg cells [29,31–33,41]. Consistent with previous researches [42], we also observed that UC-MSCs could significantly increase the frequency of Tregs in resting spleen lymphocytes (Figure 1A, 1B & 1F, *p*<0.01). In addition, we found that UC-MSCs had no effect in the stimulating and/or inhibiting the proliferation of the resting spleen lymphocytes *in vitro* (Figure 1E, *p*>0.05). However, to date, we know little whether the defective function of Tregs can be improved and how to improve the defective function of Tregs *in vitro*. It has been reported that human cord blood stem cell can modulate the defective function of Treg cells from T1D mice *in vitro* [24]. Thus, to estimate the suppressive function of Tregs, we calculated the proliferation index of PHA stimulated CFSE-labeled allogeneic spleen lymphocytes co-cultured with purified Tregs after in the presence or absence of UC-MSCs education by Modfit Soft. We found that Tregs after UC-MSCs education significantly inhibited the proliferation of PHA stimulated spleen lymphocytes *in vitro* (Figure 1C, 1D & 1G, *p*<0.01). These data indicated that the function of Tregs could be improved or corrected *in vitro* by UC-MSCs education. In addition, we observed that Tregs after UC-MSCs education exerted significantly immunosuppressive function or anti-inflammatory effect *in vivo via* decreasing the level of IFN-γ (pro-inflammatory factor) and increasing the levels of IL-10 and TGF-β1 (anti-inflammatory factors) in the peripheral plasma, compared to those mice receiving PBS treatment (Figure 2A–2C, p<0.01). These data indicated that the suppressive function of Tregs after UC-MSCs education was significantly improved *in vitro* as well as *in vivo*. In consistent with previous studies [24], the function of Tregs could be ‘‘educated’’ by the favorable microenvironment created by UC-MSCs. More importantly, we for the first time indicated that UC-MSCs also could improve the suppressive function of Tregs by co-culture *in vitro*.

Some researches indicated that systemic inflammation may be associated with the progress of the central nervous systemic inflammation [43]. Microglia, which is the primary resident immune cell of the central nervous system and considered to be in quiescent state in the healthy brain, secrete inflammatory cytokines from the resting phenotype to the activated phenotype in AD, contributing to Aβ deposition and the pathogenesis [44,45]. Ramiﬁed microglia considered to be the resting microglia were conversed into an ameboid macrophage-like morphology under pathological environment, which considered to be activated and secret the proinflammatory mediators [46]. Accompanied with the decreased systemic inflammation, we observed that the number of activated microglia by IBA-1 staining was significantly decreased in the brain receiving purified and educated Tregs treatment compared to those receiving PBS treatment (Figure 3A–3C, *p<0.05). Aβ deposition, which dues to the imbalance of biogenesis and clearance of Aβ protein, is considered to be the initial reason attributed to the neuropathology and cognitive decline of AD [47]. Thioflavin-S staining data and ELISA data revealed that transplantation of purified and educated Tregs not only reduced the size of Aβ plaque area but also the levels of soluble Aβ1-40 and Aβ1-42 in the brain (Figure 3D–3K, *p<0.05, **p<0.01). More importantly, our data indicated that systemic transplantation of autologous Tregs could significantly improve the ability of learning and memory (Figure 4A–4E, *p<0.05). Consistent with previous studies [1,9,48], our data revealed that inhibition of activated microglia and decreased levels of Aβ maybe, at least partially, associated with improved cognitive deficit of AD animal model. However, the exact mechanism about how systemic transplantation of Tregs resulted in the changes of microglia and Aβ levels is not clear to date, which deserved further investigation.

In comparison with using MSCs for treating AD, application of purified autologous Tregs from spleens after UC-MSCs education *in vitro* has some unique advantages, which include no immune rejection and shortage of suitable donor. In addition, the method for systemic transplanting Tregs is safe and easy to perform. In conclusion, transplantation of purified autologous Tregs after UC-MSCs education *in vitro* may be a more desirable alternative and attractive way to prevent the progress of AD.
